# 1,3-Thiazine, 1,2,3,4-Dithiadiazole, and Thiohydrazide Derivatives Affect Lipid Bilayer Properties and Ion-Permeable Pores Induced by Antifungals

**DOI:** 10.3389/fcell.2020.00535

**Published:** 2020-06-30

**Authors:** Anastasiia A. Zakharova, Svetlana S. Efimova, Valeriy N. Yuskovets, Igor P. Yakovlev, Zara M. Sarkisyan, Olga S. Ostroumova

**Affiliations:** ^1^Laboratory of Membrane and Ion Channel Modeling, Institute of Cytology, Russian Academy of Sciences, Saint Petersburg, Russia; ^2^Department of Organic Chemistry, Saint-Petersburg State Chemical Pharmaceutical University, Saint Petersburg, Russia; ^3^Department of General and Medical Chemistry, Saint-Petersburg State Pediatric Medical University, Saint Petersburg, Russia

**Keywords:** dithiadiazoles, thiazines, thiohydrazides, lipid bilayers, antifungals, ion-permeable pores, membrane dipole potential, membrane curvature stress

## Abstract

Over the past decade, thiazines, thiadiazoles, and thiohydrazides have attracted increasing attention due to their sedative, antimicrobial, antiviral, antifungal, and antitumor activities. The clinical efficacy of such drugs, as well as the possibility of developing resistance to antimicrobials, will depend on addressing a number of fundamental problems, including the role of membrane lipids during their interaction with plasma membranes. The effects of the eight 1,3- thiazine-, 1,2,3,4- dithiadiazole-, and thiohydrazide-related compounds on the physical properties of model lipid membranes and the effects on reconstituted ion channels induced by the polyene macrolide antimycotic nystatin and antifungal cyclic lipopeptides syringomycin E and fengycin were observed. We found that among the tested agents, the fluorine-containing compound *N*′-(3,5-difluorophenyl)-benzenecarbothiohydrazide (C6) was the most effective at increasing the electric barrier for anion permeation into the hydrophobic region of the membrane and reducing the conductance of anion-permeable syringomycin pores. A decrease in the membrane boundary potential with C6 adsorption also facilitated the immersion of positively charged syringomycin molecules into the lipid bilayer and increases the pore-forming ability of the lipopeptide. Using differential scanning microcalorimetry, we showed that C6 led to disordering of membrane lipids, possibly by potentiating positive curvature stress. Therefore, we used C6 as an agonist of antifungals forming the pores that are sensitive to membrane curvature stress and lipid packing, i.e., nystatin and fengycin. The dramatic increase in transmembrane current induced by syringomycin E, nystatin, and fengycin upon C6 treatment suggests its potential in combination therapy for treating invasive fungal infections.

## Introduction

The growth trend of fungal diseases in the etiology of hospital-acquired and some community-acquired infections (both superficial and severe visceral mycoses associated with HIV infection and hematologic diseases) and development of pathogen resistance to existing drugs due to the widespread use of broad-spectrum antibiotics and immunosuppressants requires the identification of fungi species that were previously considered non-pathogenic and new antifungal drugs and formulations. The limited antifungal armamentarium includes polyene macrolides that bind ergosterol in the fungi plasma membrane (Baginski et al., 2002, 2005; Baginski and Czub, 2009; Laniado-Laborin and Cabrales-Vargas, 2009); azoles that block ergosterol synthesis (Haller, 1985; Shapiro et al., 2011; Niwa et al., 2014); echinocandins that inhibit the synthesis of the cell wall component, β-(1,3)-D-glucan (Douglas, 2001; Kontoyiannis and Lewis, 2002; Groll et al., 2005; Wiederhold and Lewis, 2007; Ponton, 2008; Quindos et al., 2009; Sucher and Chahine, 2009); and flucytosine derivatives that inhibit purine and pyrimidine uptake and DNA and RNA synthesis (Wang et al., 1998; Rehemtulla et al., 2004; Nishi et al., 2009), and they are mainly used in combination with the polyene macrolide antibiotic amphotericin B (Keele et al., 2001; Schwarz et al., 2006; Deng et al., 2016; Molloy et al., 2018). Although azoles have obvious benefits, such as low cost, limited toxicity, and oral administration, the resistance of pathogenic fungi strains to the most commonly applied azoles, e.g., fluconazole, itraconazole, voriconazole, and posaconazole, is a serious problem (Kontoyiannis and Lewis, 2002; Fera et al., 2009). Drugs from the group of echinocandins that target *Candida* spp. and *Aspergillus genera* are ineffective in fighting many other classes of fungi, and the appearance of strains with reduced sensitivity to echinocandins is an increasing problem as well (Eschenauer et al., 2007). The polyene macrolide antibiotics, particularly the abovementioned amphotericin B (AmB), have been used for the longest time as a first-line of defense in the treatment of severe mycoses. Despite the long-term application of AmB, strains with acquired resistance to this antibiotic rarely occur due to its direct action on fungal membrane integrity. A widely recognized mechanism of AmB action includes ergosterol binding and pore formation, which lead to increased membrane permeability to ions and small organic molecules and cell death (Kasumov et al., 1979; Baginski et al., 2002; Romero et al., 2009; Cohen, 2010). Unfortunately, systemic treatment with AmB is associated with severe side effects, including nephrotoxicity and hepatotoxicity; thus, its less toxic lipid-associated formulations are used.

Combination therapy of well-known antifungal antibiotics with structurally diverse compounds that show synergistic interactions is an effective method of minimizing antibiotic toxicity and preventing the resistance of pathogenic strains. In this respect, the promising antifungal activity of thiadiazole, thiazine, and thiohydrazide derivatives (el-Shaaer et al., 1997; Matysiak et al., 2000; Magdolen et al., 2000; Vicentini et al., 2002; Singh and Kaushik, 2008) has attracted more attention. In particular, Chudzik et al. (2019) demonstrated that 4-(5-methyl-1,3,4-thiadiazole-2-yl)benzene-1,3-diol shows strong synergistic interaction with AmB that significantly reduced the antibiotic concentration required for 100% inhibition of the growth of pathogenic fungi *in vitro*. Moreover, synergistic interactions were noted for strains with reduced sensitivity to AmB and azole-resistant isolates. The authors suggested that the synergistic interaction includes facilitating the penetration of AmB through the fungal membrane by the 1,3,4-thiadiazole derivative, which disrupts the cell wall integrity.

The measurements using model lipid systems indicated the significant role of various thiadiazoles in modulating the physical properties of membranes. Kluczyk et al. (2016) showed that the above-mentioned 1,3,4-thiadiazole derivative 4-(5-methyl-1,3,4-thiadiazole-2-yl)benzene-1,3-diol and its analog with a shorter alkyl substituent 4-(5-heptyl-1,3,4-thiadiazole-2-yl)benzene-1,3-diol strongly affect the phase transition of 1,2-dipalmitoyl-sn-glycero-3-phosphatidylcholine (DPPC). The differential scanning calorimetry (DSC) data indicated that the two 1,3,4-thiadiazoles enhanced liposomal membrane fluidity by shifting the temperature of the main phase transition of DPPC toward lower values. Infrared spectroscopy measurements showed that 4-(5-heptyl-1,3,4-thiadiazole-2-yl)benzene-1,3-diol interacts with both the hydrophobic and hydrophilic regions of the lipid bilayer while 4-(5-methyl-1,3,4-thiadiazole-2-yl)benzene-1,3-diol only interacts with the hydrophilic region of the membrane. The incorporation into DPPC membranes of the fluorine-containing derivative 2-(4-fluorophenylamino)-5-(2,4-dihydroxybenzeno)-1,3,4-thiadiazole in amounts smaller than 1 mol% leads to an increase in the main phase transition temperature of DPPC (Kamiński et al., 2012). Higher concentrations of the compound lead to formation of the complex with DPPC. Gagos (2008) used a monomolecular layer technique, FTIR spectroscopy and linear dichroism-FTIR and ascertained that the chlorine-containing derivative 2-(2,4-dihydroxylphenyl)-5,6-dichlor-1,3-benzothiazole exerts a pronounced ordering effect with respect to the 1,2-diphytanoyl-*sn*-glycero-3-phosphocholine (DPhPC) acyl chains. The non-steroidal anti-inflammatory 1,2-thiazine derivatives, in particular, meloxicam, piroxicam, and tenoxicam, demonstrate the perturbing effect on the lipid membranes summarizing in a lowering the main phase transition temperature and cooperativity, and increasing the mean area per DPPC molecule (Kyrikou et al., 2004; Nunes et al., 2011). Moreover, these compounds are able to induce fusion of lipid vesicles at low drug to lipid ratio (Chakraborty et al., 2008; Majumdar et al., 2015). And the fusogenic property of the drugs, which depends on the liposome composition (Mondal Roy et al., 2010; Mondal Roy and Sarkar, 2011; Majumdar and Sarkar, 2016), is related to their perturbing effect (Mondal and Sarkar, 2009).

The aim of this study was to investigate the effects of eight new 1,3- thiazine-, 1,2,3,4- dithiadiazole-, and thiohydrazide-related compounds (Table 1) on the physical properties of model lipid membranes and reconstituted ion channels produced by known antifungals with the pore-forming mechanism of action, namely, the polyene macrolide antibiotic nystatin (Nys) and cyclic lipopeptides syringomycin E (SyrE) and fengycin (Fen).

**TABLE 1:** Chemical structure of the tested 1,3-thiazine, 1,2,3,4-dithiadiazole, and thiohydrazide derivatives.

Compound	Chemical structure	Chemical name	References*
C1	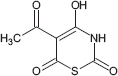	5-acetyl-4-hydroxy-2*H*-1,3-thiazine-2,6-dione	Yuskovets et al., 2007
C2	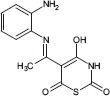	5-[*N*-(2-aminophenyl)ethanimidoyl]-4-hydroxy-2*H*-1,3-thiazine-2,6-dione	Yuskovets et al., 2006
C3	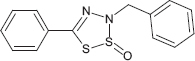	3-benzyl-5-phenyl-3H-1,2,3,4-dithiadiazole-2-oxide	Yuskovets et al., 2018
C4	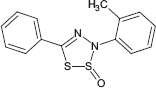	3-(2-methylphenyl)-5-phenyl-3H-1,2,3,4-dithiadiazole-2-oxide	
C5	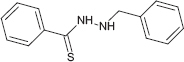	2-benzylthio-benzhydrazide	Forsgren and Sandstrom, 1960
C6	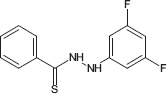	*N*′-(3,5-difluorophenyl)-benzenecarbothiohydrazide	Jensen et al., 1961
C7	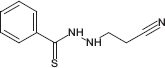	*N*′-(2-cyanoethyl)-benzenecarbothiohydrazide	
C8	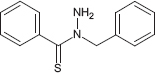	*N*-benzylbenzene-carbothiohydrazide	Forsgren and Sandstrom, 1960

*The compound was synthesized as described in References.

## Materials and Methods

### Chemicals

All chemicals used were of reagent grade. Synthetic DPPC, DPhPC, 1-palmitoyl-2-oleoyl-*sn*-glycero-3-phosphocholine (PO PC), 1-palmitoyl-2-oleoyl-*sn*-glycero-3-phospho-(1′-rac-glycer ol) (POPG), 1-palmitoyl-2-oleoyl-*sn*-glycero-3-phosphoethan olamine (POPE), 1,2-dipalmitoyl-sn-glycero-3-phospho-(1′-rac-glycerol) (DPPG), cholesterol (CHOL), and ergosterol (ERG) were obtained from Avanti^®^ Polar Lipids. Non-actin, KCl, HEPES, DMSO, nystatin (Nys), and fengycin (Fen) were purchased from Sigma-Aldrich Company Ltd. (Gillingham, United Kingdom).

The chemical names and structures of the tested 1,3- thiazine-, 1,2,3,4- dithiadiazole-, and thiohydrazide-related compounds and references with a description of the synthesis methods are presented in Table 1.

KCl solutions (0.1 or 2.0 M) were buffered using 10 mM HEPES-KOH at pH 7.4 or 10 mM CHES-KOH at pH 9. Syringomycin E (SyrE) was isolated and purified as described previously (Bidwai and Takemoto, 1987), and it was kindly offered by Dr. J. Y. Takemoto (Utah State University, United States). All experiments were performed at room temperature (25°C).

### Membrane Boundary Potential Measurements

Virtually solvent-free planar lipid bilayers were prepared using a monolayer-opposition technique (Montal and Muller, 1972) using a 50-μm diameter aperture and a 10-μm thick Teflon film separating the two (*cis*- and *trans*-) compartments of the Teflon chamber. The aperture was pretreated with hexadecane. Lipid bilayers were made from pure DPhPC, pure POPC, and an equimolar mixture of POPC and POPG. The steady-state conductance of K^+^-non-actin was modulated via the two-sided addition of the tested 1,3- thiazine-, 1,2,3,4- dithiadiazole-, and thiohydrazide-related compounds from different mM stock solutions in DMSO to the membrane-bathing solution (0.1 M KCl, pH 7.4) to obtain a final concentration ranging from 5 μM to 1 mM. Ag/AgCl electrodes with 1.5% agarose/2 M KCl bridges were used to apply *V* and measure the transmembrane current. The “positive voltage” refers to the case in which the *cis*-side compartment is positive with respect to the *trans*-side. The current was measured using an Axopatch 200B amplifier (AutoMate Scientific Inc., Berkeley, CA, United States) in the voltage clamp mode. Data were digitized using a Digidata 1440A and analyzed using pClamp 10.0 (AutoMate Scientific Inc., Berkeley, CA, United States) and Origin 8.0 (OriginLab Corporation, Northampton, MA, United States). Data were acquired at a sampling frequency of 5 kHz using low-pass filtering at 200 Hz, and the current tracks were processed through an 8-pole Bessel 100-kHz filter.

The conductance of the lipid bilayers was determined by measuring *I* at a constant transmembrane voltage (*V* = 50 mV). In the subsequent calculations, the membrane conductance (*G*) was assumed to be related to the membrane boundary potential (φ_*b*_), the potential drop between the aqueous solution and the membrane hydrophobic core by the Boltzmann distribution (Andersen et al., 1976):

(1) $$G∼\xi \cdot C\,\exp\left(-\frac{\mathrm{ze}\,\phi_{\text{b}}}{\mathrm{kT}}\right)$$

where *ξ* is the ion mobility, *ze* is the ion charge, *k* is the Boltzmann constant, and *T* is the absolute temperature.

A Langmuir adsorption isotherm was used to describe the adsorption of the tested 1,3- thiazine-, 1,2,3,4- dithiadiazole-, and thiohydrazide-related compounds to lipid bilayers in a first-order approximation as follows (de Levie et al., 1979; Reyes et al., 1983; Cseh et al., 2000; Efimova and Ostroumova, 2012; Ostroumova et al., 2013):

(2) $$\Delta \,\phi_{\text{b}}\,\left(C\right)=\frac{\Delta \,\phi_{\text{b}}\,\left(\infty \right)\,C}{C+K},$$

where the Δφ_*b*_(*C*) is the boundary potential change at the *C* concentration of the compound, Δφ_*b*_(*∞*) is the maximum potential change, and *K* is the desorption constant, which provides a meaningful measure of the affinity between the agent and the lipid phase. The desorption constant can be determined as the slope of the linear dependence of [Δφ_*b*_(∞)]/[Δφ_*b*_(*C*)] on 1/*C*. The linear approximation of the indicated dependences was performed using Origin 8.0 (Origin Lab).

### Differential Scanning Microcalorimetry

Differential scanning calorimetry experiments were performed by a μDSC 7EVO microcalorimeter (Setaram, France). Giant unilamellar vesicles were prepared from pure DPPC and DPPG or mixtures of 90 mol% DPPC and 10 mol% CHOL (DPPC/CHOL), and 40 mol% DPPC, 50 mol% DPPG and 10 mol% ERG (DPPC/DPPG/ERG) by the electroformation method (standard protocol, 3 V, 10 Hz, 1 h, 55°C). The liposome suspension contained 5 mM lipid and was buffered by 5 mM HEPES-KOH at pH 7.4. The tested 1,3- thiazine-, 1,2,3,4- dithiadiazole-, and thiohydrazide-related compounds were added to aliquots to obtain a lipid:agent molar ratio of 50:1 and 10:1. The liposomal suspension was heated at a constant rate of 0.2 C⋅min^–1^. The reversibility of the thermal transitions was assessed by reheating the sample immediately after the cooling step from the previous scan. The temperature dependence of the excess heat capacity was analyzed using Calisto Processing (Setaram, France). The thermal behavior of the lipids in the absence and presence of the tested 1,3- thiazine-, 1,2,3,4- dithiadiazole-, and thiohydrazide-related compounds was described by the changes in the temperature of the pretransition peak (Δ*T*_*p*_), the maximum temperature of the main phase transition (Δ*T*_*m*_), the half-width of peak in the endotherm (Δ*T*_1/2_), and the changes in the enthalpy of the main phase transition (ΔΔ*H*).

### Reconstitution of Ion Channels Into Planar Lipid Bilayers

Using Montal and Muller technique (1972) lipid bilayers were made from DPhPC, a mixture of 67 mol% DPhPC and 33 mol% CHOL, and a mixture of 20 mol% POPC, 20 mol% POPE, 50 mol% POPG and 10 mol% ERG. After the membrane was completely formed, stabilized stock solutions of SyrE, Nys, and Fen (in water at pH 3.0, DMSO, and methanol, respectively) were added to the aqueous phase on the *cis*-side of the bilayer to obtain a final concentration ranging from 0.5 to 10.0 μM of SyrE, 7–20 μM of Nys and 4–8 μM of Fen. Lipid bilayers were bathed in 2.0 M KCl at pH 7.4 (study of single SyrE channels and pore forming ability of Nys), 0.1 M KCl at pH 7.4 (measurements of SyrE induced steady-state transmembrane current), and 2.0 M KCl at pH 9.0 (study of pore forming ability of Fen). The tested 1,3- thiazine-, 1,2,3,4- dithiadiazole-, and thiohydrazide-related compounds were added to both sides of the membrane up to 100 or 400 μM.

Single-channel conductance (*g*) was defined as the ratio between the current flowing through a single channel (*i*) and *V*. The conductance fluctuation and dwell time (τ) histograms were made for the constant transmembrane voltages. The total number of events used for the channel amplitude and dwell time analysis was 800–1,000 and 1,500–3,000, respectively. Peaks on the conductance and dwell time histograms were fitted by the normal density and exponential functions, respectively. The χ^2^ criterion was applied (*P* < 0.05). The probability of the channel to be in an open state (*P*_*op*_) was determined as $\frac{\tau }{\tau +\tau_{\text{close}}}$, where τ is the dwell time of the single channel and τ_*close*_ is the time that the channel is in a closed state. The *P*_*op*_-values were averaged from 150 to 200 single channel bursts (mean ± sd).

A steady-state antifungal-induced transmembrane current (*I*_∞_) was used to assess the channel-forming activity of polyene macrolide and lipopeptides after and before two-sided additions of the tested 1,3- thiazine-, 1,2,3,4- dithiadiazole-, and thiohydrazide-related compounds. The mean ratios (*I*_∞_/*I*_∞_^0^) of the macroscopic currents after (*I*_∞_) and before (*I*_∞_^0^) two-sided modifier addition were averaged from 5 to 7 bilayers (mean ± sd).

## Results and Discussion

### Effects of 1,3-Thiazine, 1,2,3,4-Dithiadiazole, and Thiohydrazide Derivatives on the Electrical Properties of Lipid Bilayers and Transport Through Ion-Selective Pores

**FIGURE 1:**
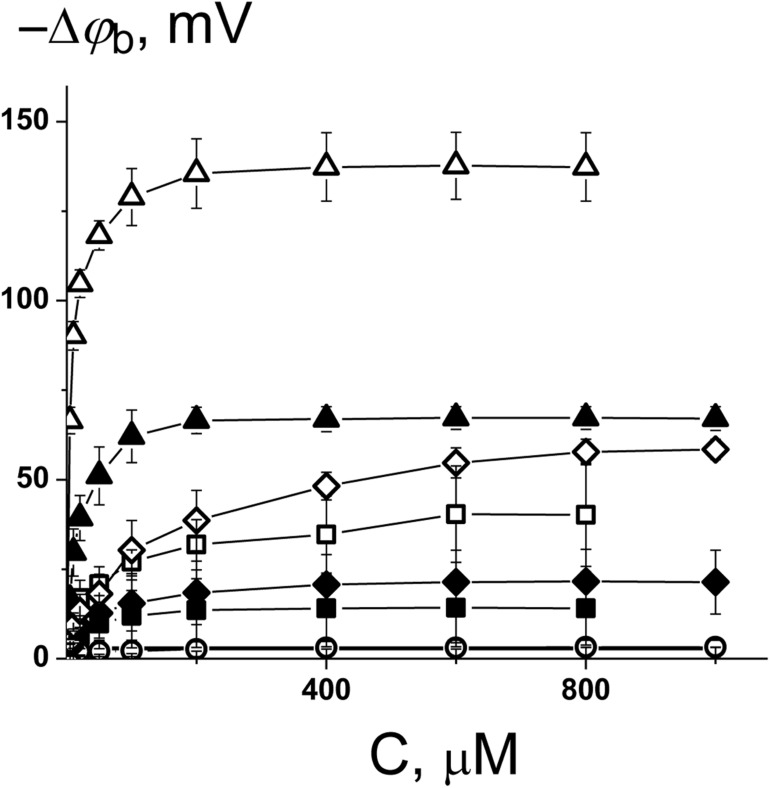
Dependence of the decrease in the boundary potential of the membrane (–Δφ_*b*_) on the concentration of C1 (■), C2 (□), C3 (•), C4 (○), C5 (▲), C6 (Δ), C7 (♦), and C8 (◊). The membranes were composed of DPhPC and bathed in 0.1 M KCl at pH 7.4. *V* = 50 mV.

To ascertain the changes in the distribution of electrical potentials on the membrane-solution interface upon the adsorption of the 1,3- thiazine-, 1,2,3,4- dithiadiazole-, and thiohydrazide-related compounds, a method based on the modulation of the non-actin-induced steady-state transmembrane current by the tested agents has been applied. Figure 1 shows the dependences of the boundary potential of DPhPC membranes on the concentration of the tested derivatives C1–C8. The curves presented in Figure 1 are close to linear at low agent concentrations and tend toward saturation at high concentrations. A Langmuir adsorption isotherm has been used to describe the adsorption of the 1,3-thiazine, 1,2,3,4-dithiadiazole, and thiohydrazide derivatives to lipid bilayers in a first-order approximation. Table 2 shows the characteristic parameters of the Langmuir adsorption isotherm, namely, the maximum changes in the φ_*b*_ of the DPhPC membranes at an infinitely high concentration of the tested derivatives C1–C8 [−Δφ_*b*_(max)], and their desorption constants (*K*); thus, it provides a meaningful measure of the affinities between the agents and the lipid phase.

**TABLE 2:** Characteristic parameters of the Langmuir adsorption isotherms used to describe the adsorption of 1,3-thiazine, 1,2,3,4-dithiadiazole, and thiohydrazide derivatives on lipid bilayers with different compositions.

Compound	Membrane composition					
	DPhPC		POPC		POPC/POPG	
	−Δφ_*b*_(max), mV	*K*, μ M	−Δφ_*b*_(max), mV	*K*, μ M	−Δφ_*b*_(max), mV	*K*, μ M
C1	14 ± 10	25 ± 9	20 ± 8	69 ± 18	−	−
C2	40 ± 12	42 ± 12	60 ± 10	57 ± 4	44 ± 14	23 ± 5
C3	1 ± 1	−^$^	7 ± 5	−^$^	17 ± 4	4 ± 3
C4	3 ± 1	−^$^	5 ± 3	−^$^	−	−
C5	67 ± 8	15 ± 3	49 ± 15	24 ± 8	50 ± 10	15 ± 4
C6	137 ± 10	6 ± 1	100 ± 25	10 ± 2	14 ± 6	22 ± 7
C7	21 ± 9	28 ± 8	20 ± 9	77 ± 14	−	−
C8	60 ± 8	60 ± 10	60 ± 12	49 ± 15	50 ± 7	100 ± 15

**Δφ_*b*_(max), the maximum change in the membrane boundary potential; and K, desorption constant*. *$, desorption constant is not determined.**

One can see that C6 demonstrates the pronounced efficiency to reduce the boundary potential of the DPhPC membranes (−Δφ_*b*_ exceeds 100 mV); C5 and C8 are characterized by values that are at least a half of the C6 effect (approximately 50 mV); C1, C2, and C7 present insignificant effects (Δφ_*b*_ does not exceed 30 mV); C3 and C4 have practically no effect on the φ_*b*_-magnitude (Figure 1 and Table 2). Table 2 and Supplementary Figure S1A demonstrate the similarity of 1,3-thiazine, 1,2,3,4-dithiadiazole, and thiohydrazide derivatives on membranes composed of DPhPC and POPC.

A comparison of the structures and φ_*b*_-modifying abilities of the tested compounds led to several important observations. (*i*) The C5 and C8 molecules have high structural similarity and present two benzene rings bound by a sufficiently flexible linker containing heteroatoms, and they have significant potential-modifying ability, which is likely due to their high hydrophobicity and polarity (high values of the logarithm of the octanol/water partition coefficient, LogD_*o/w*_, and high magnitudes of the dipole moment, μ, Supplementary Table S1). (*ii*) The inclusion of the fluorine atoms into the modifier molecule promotes a dramatic ability to influence the boundary potential, which should be related to a shift in the electron density upon the introduction of halogen substituents with high electronegativity (C6 vs. C5) and the highest partition coefficient among tested compounds (Supplementary Table S1). (*iii*) The 1,3-thiazine-related compounds C1 and C2 have a similar structural core of a heterocyclic nature, and the small boundary potential change in their presence is most likely due to high hydrophilicity of 1,3-thiazine derivatives (low LogD_*o/w*_-values, Supplementary Table S1). The greater efficiency of C2 might be related to its higher partition coefficient and dipole moment compared to C1 (Supplementary Table S1). (*iv*) The 1,2,3,4-dithiadiazole-related compounds C3 and C4 have two benzene rings connected by a relatively rigid heterocycle linker and are not characterized by the ability to influence the boundary potential of the membrane despite their high hydrophobicity and significant polarity (Supplementary Table S1). This may indicate the importance of not so much the magnitude of the molecule dipole moment as its orientation in the bilayer.

The electric field distribution at the boundaries of the membrane consists of diffuse part of the electrical double layer and the potential drop over polar area inside the membrane itself. The latter is generally attributed to the dipole effect, which is related to specific orientation of lipid and water dipoles and called membrane dipole potential, φ_*d*_, which depends on the lipid hydration and phase state (Nesterenko and Ermakov, 2012). It should also be noted that the adsorption of the charged molecules can affect both the first and second components. In particular, the adsorption of the Gd^3+^ leads to a dramatic decrease in the dipole component of the boundary potential (Ermakov and Yusipovich, 2002). To determine whether the charged or uncharged form of the tested molecules modulates the boundary potential, we tested negatively charged bilayers composed of POPC/POPG as well as in neutral membranes made from DPhPC or POPC (Figure 1, Supplementary Figure S1A and Table 2). Supplementary Figure S1B demonstrates the concentration dependences of the decrease in the φ_*b*_ of POPC/POPG-membrane in the presence of 1,3-thiazine, 1,2,3,4-dithiadiazole, and thiohydrazide derivatives and shows that almost all compounds are characterized by effects similar to those observed in neutral membranes. However, the most effective compound in the case of DPhPC and POPC bilayers, i.e., C6, has almost no effect on the φ_*b*_ of membranes composed of POPC/POPG. This finding suggests that the negatively charged form of C6, which is not adsorbed on the negatively charged membranes, changes the bilayer boundary potential of the DPhPC and POPC bilayers. However, these data do not answer the question of whether the surface or dipole component of the membrane boundary is changed by C6 adsorption.

The pronounced φ_*b*_-modifying ability of C6 and C8 indicates their potential in applications for modulating the functioning of ion-selective pores that are known to be sensitive to the distribution of the electric potential at the membrane-solution interface. The antifungal effects of cyclic lipopeptide produced by the plant bacterium *Pseudomonas syringae* pv. *syringae*, SyrE, are reported to be related to pore formation in the host membrane leading to cytolysis (Hutchison and Gross, 1997; Bender et al., 1999; Dalla Serra et al., 1999). It was shown that SyrE forms asymmetric peptide-lipid pores of a conical shape with predominant anion selectivity (Malev et al., 2002; Ostroumova et al., 2007a). The properties of single SyrE pores and lipopeptide induced steady-state transmembrane current depend on the boundary potential of membrane (Feigin et al., 1996; Schagina et al., 1998; Dalla Serra et al., 1999; Ostroumova et al., 2005, 2007b, 2008; Efimova et al., 2018b). A previous study showed that a decrease in the membrane dipole potential upon addition of small molecule modifiers leads to a reduction of SyrE-pore conductance and dwell time and an increase in the SyrE-induced steady-state transmembrane current (Ostroumova et al., 2007b, 2008; Efimova et al., 2018b). The effect on multichannel activity is due to the facilitation of the immersion of positively charged SyrE molecules into the lipid bilayer with a decrease in its dipole potential (Ostroumova et al., 2008; Efimova et al., 2018b).

**FIGURE 2:**
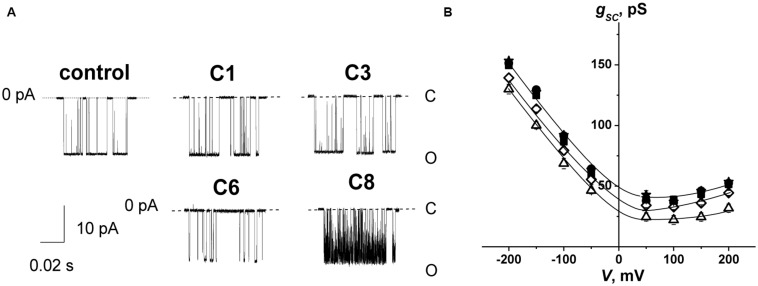
**(A)** Current fluctuations corresponding to the openings and closings of single SyrE channels in the absence (*control*) and presence of 100 μM of C1, C3, C6, and C8. *V* = –200 mV. *C*, closed state of the channel, and *O*, open state of the pore. **(B)** *G*(*V*) curves of single SyrE channels in the absence (■) and presence of 100 μM of C1 (■), C3 (•), C6 (Δ), and C8 (◊). The membranes were composed of DPhPC and bathed in 2.0 M KCl at pH 7.4.

To test the influence of the 1,3-thiazine, 1,2,3,4-dithiadiazole, and thiohydrazide derivatives on SyrE pores, we used two compounds that are practically ineffective in terms of the boundary potential, i.e., C1 and C3, as well as C6 and C8. Figure 2A shows examples of current fluctuations corresponding to the opening and closure of single SyrE channels in DPhPC bilayers bathed in 2 M KCl in the absence (control) and presence of 100 μM of C1, C3, C6, and C8 at a transmembrane voltage of −200 mV. The addition of C6 and C8 slightly decreases the channel amplitude, while the addition of C1 and C3 has almost no effect on the pore conductance. Figure 2B presents the conductance-voltage curves of the SyrE channels in the absence and presence of 1,3-thiazine, 1,2,3,4-dithiadiazole, and thiohydrazide derivatives. Table 3 demonstrates that C6 and C8 reduce the SyrE channel conductance by approximately 15 and 10%, respectively, while C1 and C3 do not influence this parameter at all. The Debye radius is small and the effects of the surface component of the boundary potential should be discarded in solutions of high ionic strength (2 M). Thus, one can conclude that the dipole component and not the surface component of the boundary potential has been altered by C6 and C8 adsorption onto lipid bilayers. The effects of C6 and C8 on single SyrE pore amplitudes should be related to the anion selectivity of these channels (Takemoto et al., 2003): the decreased dipole potential (i.e., virtual plus in the hydrophobic interior) upon C6 and C8 introduction leads to an increase in the electric barrier for anion permeation into the hydrophobic region of the membrane, which causes a reduction in the conductance of anion-permeable SyrE-pores.

**TABLE 3:** Effects of 1,3-thiazine, 1,2,3,4-dithiadiazole, and thiohydrazide derivatives on the ion-permeable pores induced by the different antifungals SyrE, Fen, and Nys.

Compound	Antifungal pore-forming agent					
	SyrE*				Nys^&^	Fen^#^
		
	*g_*sc*_, pS*	*τ, ms*	*P*_*op*_	*I*_∞_*/I*_∞_^0^	*I*_∞_*/I*_∞_^0^	
–	153 ± 2	126 ± 20	0.8 ± 0.2	−	−	−
C1	150 ± 3	96 ± 16	0.8 ± 0.3	0.9 ± 0.1	0.9 ± 0.1	1.0 ± 0.1
C3	151 ± 2	98 ± 16	0.7 ± 0.2	0.5 ± 0.2	0.9 ± 0.1	0.9 ± 0.1
C6	130 ± 3	15 ± 4	0.2 ± 0.1	8.2 ± 3.7	5.6 ± 0.7	8.9 ± 3.1
C8	140 ± 3	19 ± 2	0.4 ± 0.2	0.7 ± 0.3	0.8 ± 0.1	0.8 ± 0.2

g_*SC*_, conductance of single channels in the absence and presence of the compound at V = −200 mV; τ, dwell time of single pores; I_∞_/I_∞_^0^, ratio of the transmembrane current induced by the antifungal in the presence and absence of modifier at V = 50 mV. The lipid bilayers were composed of DPhPC (*), DPhPC/CHOL (^&^), and POPC/POPE/POPG/ERG (^#^).

Figure 2A also shows that C6 and C8 induces more flickery behavior of SyrE channels than in the absence of any modifiers (control) or presence of C1 and C3. An examination of the lifetime of SyrE pores showed that C6 and C8 also led to an approximately 10-fold reduction in channel dwell time while C1 and C3 are not characterized by a significant ability to modify the SyrE-pore lifetime (Table 3). The similar trend is observed for the probability of SyrE channels to be open, *P*_*op*_ (Table 3). A possible explanation for the dramatic changes in the lifetime and open probability of SyrE channels is that the opening or closure of the SyrE channel may include the movement of the polar or charged groups of pore-forming molecules (lipopeptide or lipid) through the region of dipole potential jump (Ostroumova et al., 2007b) which is altered by C6 and C8 adsorption.

An increase in the pore-forming ability of SyrE with the addition of C6 could be caused by the promotion of the adsorption of positively charged lipopeptide molecules onto membranes by negatively charged C6 molecules and the reduction in the electric barrier for SyrE cation permeation into membrane at the decreasing φ_*d*_ by C6. Table 3 shows the validity of this assumption. The addition of 100 μM of C6 into the membrane bathing solution (0.1 M KCl) leads to an eightfold increase in the steady-state transmembrane current induced by SyrE. The obtained result suggests the synergism of the antifungal action of SyrE and C6. C1 has almost no effect on the *I*_∞_ value. Surprisingly, the introduction of C3 and C8 leads to a 1.5- to 2-fold decrease in the SyrE-induced transmembrane current (Table 3). Taking into account the different dipole-modifying ability of C3 and C8 (Figure 1 and Table 2), one can conclude that the decrease in SyrE activity is not related to an alteration of φ_*d*_. The effects might be associated with the influence of the tested derivatives on the elastic properties of the membrane, to which SyrE pores with a positively curved lipid mouth are also sensitive (Malev et al., 2002; Ostroumova et al., 2007a).

### Effect of 1,3-Thiazine, 1,2,3,4-Dithiadiazole, and Thiohydrazide Derivatives on the Lipid Packing and Ion Channels Sensitive to Transbilayer Pressure Profile

**FIGURE 3:**
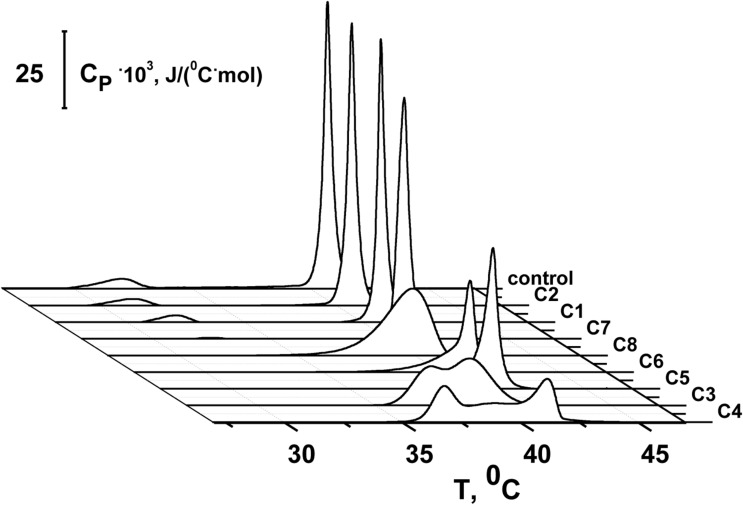
Heating DSC thermograms of DPPC liposomes in the absence (*control*) and presence of 1,3-thiazine (C1 and C3), 1,2,3,4-dithiadiazole (C3 and C4), and thiohydrazide derivatives (C5–C8) at a lipid:compound molar ratio of 10:1.

To study the effects of 1,3-thiazine, 1,2,3,4-dithiadiazole, and thiohydrazide derivatives on the phase behavior of the membrane-forming lipids, we performed DSC of large unilamellar DPPC vesicles at different lipid:compound (*L:C*) molar ratios. Figure 3 shows the typical DSC heating thermograms of DPPC liposomes in the absence (*control*) and presence of 1,3-thiazine, 1,2,3,4-dithiadiazole, and thiohydrazide derivatives at a *L:C* molar ratio of 10:1. The DSC curve of DPPC vesicles in the absence of any modifiers (*control*) is characterized by two well-defined transitions. The transition from the lamellar gel phase to the rippled gel phase, named the pretransition, occurs at approximately 34°C and it is due to a rearrangement of the polar head groups of DPPC. The second (main) transition, which is from the rippled gel phase to the liquid phase, is observed at 41.2°C and due to the melting of palmitoyl chains (Koynova and Caffrey, 1998). The changes induced by the presence of the 1,3-thiazine, 1,2,3,4-dithiadiazole, and thiohydrazide derivatives on the thermotropic properties of these transitions offer the opportunity to investigate the effect of these compounds on two distinct regions of the lipid bilayer. Table 4 reports the changes in the maximum temperatures of the pretransition (Δ*T*_*p*_) and the main transition (Δ*T*_*m*_), the half-width of the main peak (Δ*T*_1/2_), and the enthalpy of the main phase transition of DPPC on increasing compound concentration from *L:C* molar ratio of 50:1 to 10:1. Figure 3 and Table 4 show that C1 has no effect while C2 and C7 demonstrate only a small effects on the DPPC thermotropic properties independently on *L:C* ratio, suggesting that these compounds weakly interact with the DPPC bilayer at the surface level and do not significantly perturb the lipid organization in the membrane. Moreover, C2 and C7 affect the pretransition but not the main transition of DPPC. The modifications of the pretransition in the presence of C2 and C7 (the pretransition peak is shifted by C2 toward a lower temperature by approximately 0.5°C and suppressed by C7) indicates the interaction of these compounds with the polar lipid head group region.

**TABLE 4:** Parameters that characterize the thermotropic behavior of DPPC in the presence of 1,3-thiazine, 1,2,3,4-dithiadiazole, and thiohydrazide derivatives in liposome suspension at different lipid:compound (*L:C*) molar ratios.

Compound	*L*:*C* ratio	DSC parameters			
		Δ *T*_*p*_, °C	*−*Δ *T*_*m*_, °C	Δ *T*_1/2_, °C	−Δ Δ *H*, kcal/mol
C1	*50:1*	0	0	0	5 ± 3
	*10:1*	0	0	0	6 ± 3
C2	*50:1*	0.5 ± 0.3	0	0	5 ± 3
	*10:1*	0.5 ± 0.2	0	0	5 ± 3
C3	*50:1*	−*	0.7 ± 0.2	0.8 ± 0.3	10 ± 3
	*10:1*	−*	1.0 ± 0.2	3.1 ± 0.3	8 ± 3
C4	*50:1*	−*	0.4 ± 0.2	1.0 ± 0.2	9 ± 4
	*10:1*	−*	0.5 ± 0.1	4.2 ± 0.7	7 ± 2
C5	*50:1*	−*	0.2 ± 0.1	0.1 ± 0.1	7 ± 4
	*10:1*	−*	0.2 ± 0.1	0.1 ± 0.1	7 ± 4
C6	*50:1*	−*	0.2 ± 0.1	0.2 ± 0.1	16 ± 4
	*10:1*	−*	0.2 ± 0.1	0.2 ± 0.1	13 ± 4
C7	*50:1*	−*	0	0	4 ± 2
	*10:1*	−*	0.1 ± 0.1	0.1 ± 0.1	3 ± 2
C8	*50:1*	−*	0.5 ± 0.1	0.5 ± 0.1	8 ± 4
	*10:1*	−*	0.8 ± 0.2	2.0 ± 0.4	5 ± 3

*ΔT_*p*_, changes in the maximum temperature of the pretransition; ΔT_*m*_, changes in the maximum temperature of lipid melting; ΔT_1/2_, changes in the half-width of the main peak; and ΔΔH, enthalpy changes of the main phase transition*. *The pretransition is suppressed. The temperatures of the pretransition and main transition of untreated DPPC are approximately 34.0 and 41.2°C, respectively, with the half-width of the main peak of approximately 0.5°C. These data are in a good agreement with the results of Koynova and Caffrey (1998).

C5 and C6 suppress the pretransition and slightly affect the main transition of DPPC (the main peak is shifted toward a lower temperature by approximately 0.2°C and the half-width of the main peak is increased only by 0.1–0.2°C) independent of the *L:C* ratio. C3, C4, and C8 show that stronger effects on the DPPC bilayers induce modifications of both the pretransition and the mean transition peaks. The pretransition peak is suppressed even at low compound concentrations. The thermograms reveal that C3, C4, and C8 are able to penetrate into the bilayer hydrophobic core, disrupting the regular packing of lipid acyl chains as assessed by the dramatic modification of the main transition peak of DPPC. These compounds lead to significant broadening and a shift of the main transition peak toward lower temperatures. These effects depend on the *L:C* ratio. At an *L:C* ratio of 50:1, a slight modification of the peak is observed (−Δ*T*_*m*_ is equal to 0.4–0.7°C, Δ*T*_1/2_, is equal to 0.5–1.0°C), and at an *L:C* ratio of 10:1, a dramatic changes have occurred (−Δ*T*_*m*_ is equal to 0.5–1.0°C, Δ*T*_1/2_, is equal to 2.0–4.2°C). Furthermore, C3, C4, and C6 are able to induce the formation of domains of different *L:C* compositions as demonstrated by the presence of a multicomponent DSC profile (Supplementary Figure S2 and Supplementary Table S2). Likely, the left peak (number 1) is related to the DPPC-enriched phase while one or two right peaks on the thermograms (numbers 2 and 3) should be related to the mixed domains that contain both DPPC and the tested compound in different proportions. The largest drop in the enthalpy of the main transition observed in the case of C6 (approximately 6%) might indicate its ability to strongly influence the membrane curvature stress, which likely occurred by the promotion of the positive spontaneous curvature and formation of non-bilayer hexagonal *L:C*6-structures. These changes might be due to the asymmetry of the C6 molecule caused by the introduction of halogen substituents into only one of two benzene rings and might also be related to the electrostatic repulsion of negatively charged C6 molecules in the membrane.

The latter suggestion might be of fundamental importance in terms of the possible synergism of the action of C6 and the antifungal polyene macrolide antibiotics amphotericin and Nys. The pores formed by the one-sided introduction of these antibiotics into the membrane have a lipid mouth with a positive curvature in the opposite monolayer leaflet and low molecular weight membrane modifiers that provoke positive curvature stress might enhance the pore-forming ability of amphotericin and Nys (Kleinberg and Fninkelstein, 1984; Chulkov et al., 2015; Efimova et al., 2018a, b). To test this possibility, we have performed measurements of the steady-state Nys-induced transmembrane current before and after addition of C6. We have also tested the effects of C1, which has been shown to have no effect on lipid packing, and the impacts of the most effective (in the regard of lipid melting) agents C3 and C8.

**FIGURE 4:**
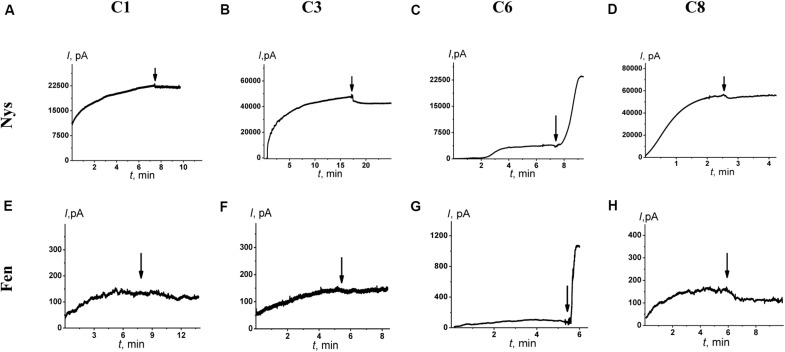
Effects of 1,3-thiazine, 1,2,3,4-dithiadiazole, and thiohydrazide derivatives on the steady-state transmembrane current induced by 7–20 μM Nys (upper panel) and 4–8 μM Fen (lower panel). The moments for the addition of 100 μM of C1 **(A,E)**, C3 **(B,F)**, C6 **(C,G)**, and C8 **(D,H)** to the bilayer-bathing solution are indicated by the arrows. The lipid bilayers were composed of DPhPC/CHOL (67/33 mol%) and bathed in 2.0 M KCl at pH 7.4 (upper panel) and POPC/POPE/POPG/ERG (20/20/50/10 mol%) and bathed in 2.0 M KCl at pH 9.0 (low panel). *V* = 50 mV.

Figures 4A–D shows the effects of 100 μM of C1, C3, C6, and C8 on the multichannel activity of Nys in the DPhPC/CHOL-membranes bathed in 2.0 M KCl. The addition of C6 leads to a significant increase in the steady-state Nys-induced transmembrane current, while the introduction of C1, C3, and C8 has almost no effect on the Nys pore-forming ability. These effects cannot be attributed to the alteration in membrane dipole potential induced by C6 and C8 due to their different action on Nys channels (Figures 4C,D) and a previously shown insensitivity of Nys pore forming ability on φ_*d*_-magnitude (Chulkov et al., 2015). More probable, the increase in Nys-induced transmembrane current is due to the altering the transbilayer pressure profile by C6 that has been found to reduce the temperature and cooperativity of phase transition of DPPC/CHOL at *L:C* molar ratio of 10:1 (−Δ*T*_*m*_ and Δ*T*_1/2_ are equal to 0.4 ± 0.2 and 0.5 ± 0.1°C, respectively) similar to pure DPPC (Table 4). Table 3 presents the mean ratio of the Nys-provoked steady-state transmembrane currents in the presence and absence of the tested 1,3-thiazine, 1,2,3,4-dithiadiazole, and thiohydrazide derivatives (*I*_∞_*/I*_∞_^0^). C6 leads to a fivefold increase in *I*_∞_. The addition of C1, C3, and C8 does not lead to reliable changes in the *I*_∞_ value. However, a slight downward trend is still observed in the cases of C3 and C8. To confirm this trend, we have increased the concentration of these compounds up to 400 μM. At the concentration of 400 μM, C6 produce about 15-fold increase, while C3 and C8 produce a 1.3- and 4-fold decrease in the steady-state Nys-induced current through CHOL-containing membranes, respectively (data not shown). The reduction in *I*_∞_ value in the presence of C3 and C8 might indicate that the membrane disordering action of the C3 and C8 observed by DSC is related to the promotion of negative curvature stress by these compounds. Two benzene rings in the structure of both molecules can contribute to their immersion into the hydrophobic region of the membrane, an increase in the lateral pressure in this region, and the inhibition of the formation of the lipid mouth of Nys pores with positive curvature. In these terms, it is easy to explain the drop in the SyrE-induced transmembrane current upon the addition of C3 and C8 (Table 3).

It is known that the antifungal cyclic lipopeptide produced by *Bacillus subtilis*, Fen, acts by making the plasma membrane of the target cell more permeable (Vanittanakom et al., 1986; Patel et al., 2011). The Fen-induced increase in membrane permeability is related to the formation of ion channels of various conductances and non-ideal cationic selectivity (Zakharova et al., 2019). It was also shown that the alteration in membrane dipole potential does not affect the pore-forming ability of this lipopeptide while the membrane adsorption of small molecules that decreased the lipid packing density enhances the steady-state Fen-induced transmembrane current (Zakharova et al., 2019). Thus, the significant effects of C3, C6, and C8 on lipid packing (Figure 3 and Table 4) indicate their potential applications for up-regulating the pore-forming ability of Fen. Figures 4E–H demonstrates the action of 100 μM of C1, C3, C6, and C8 on the multichannel activity of Fen in the POPC/POPE/POPG/ERG-membranes bathed in 2.0 M KCl. As expected, C1 does not affect the Fen pore-forming activity (Figure 4E). C6 potentiates the pore-forming activity of Fen (Figure 4G). Surprisingly, C3 and C8 do not produce the effect similar to C6 (Figures 4F,H) despite their more pronounced action on the lipid melting (Figure 3 and Table 4). Table 3 summarizes the data obtained. The almost 10-fold increase in *I*_∞_ value in the presence of C6 can be explained by the strong dependence of the pore forming ability of Fen on the presence of negatively charged species in the bilayer (Zakharova et al., 2019) or the more pronounced disordering effect of C6 on negatively charged bilayers containing PG compared to neutral ones made from PC. The electrostatic repulsion of the negatively charged lipids and C6 molecules should cause a significant decrease in the membrane packing density impacting the oligomerization of lipopeptide molecules that form pores. To confirm this assumption, we have performed the DSC measurements with DPPG containing vesicles. A comparison of Supplementary Table S3 and Table 4 shows that C6 has a significantly greater effect on the thermotropic behavior of DPPG relative to DPPC. It should be also noted that the efficiency of C6 to affect phase behavior of the DPPC/DPPG/ERG mixture at *L:C* molar ratio of 10:1 (−Δ*T*_*m*_ and Δ*T*_1/2_ are equal to 0.8 ± 0.2 and 0.9 ± 0.3°C, respectively) is comparable to that of pure DPPG (Supplementary Table S3). Moreover, C3 and C8 are characterized by depleted efficiency and have a greater effect on the thermotropic behavior of DPPG compared to DPPC, even at a high *L:C* ratio of 10:1. In particular, the −Δ*T*_*m*_ value of DPPG upon the addition of C3 is equal to 0.5° while that for DPPC at the same C3 concentration is equal to 1.0°C, and the Δ*T*_1/2_ of DPPG is equal to the 0.7°C while that for DPPC is 3.1°C. A similar effect is observed for C8. The low disordering action of C3 and C8 on negatively charged DPPG membranes is consistent with the inability of these compounds to facilitate pore formation by Fen in lipid bilayers containing POPG (Figures 4F,H and Table 3).

A summary of the obtained data indicates that (*i*) 1,2,3,4-dithiadiazole (C3 and C4) and thiohydrazide derivatives (C5, C6, and C8) greatly affect the physical properties of lipid bilayers, including the membrane dipole potential, lipid packing, and curvature stress; (*ii*) *N*′-(3,5-difluorophenyl)-benzenecarbothiohydrazide (C6) potentiates the pore-forming activity of SyrE, Nys and Fen by modulating the interaction of these antifungal agents with the lipid matrix. This finding might open up new horizons for combination therapy. Further research is required to investigate the potential toxicity of the thiohydrazide derivative which might limit its pharmacological application. The available published data testify to low toxicity of some close analogs. For example, *in vitro* and *in vivo* experiments showed that the hydrazide derivatives can induce the death of neoplastic cells without harming healthy cells (Wandall and Tarp, 2008). Antitubercular 1,3,4-thiadiazoles demonstrated low mutagenicity and toxicity against proliferating cell lines and isolated human hepatocytes (Karabanovich et al., 2016). The leishmanicidal 1,3,4-thiadiazole derivatives showed low level of toxicities to macrophages (Poorrajab et al., 2009).

## Data Availability Statement

All datasets generated for this study are included in the article/Supplementary Material.

## Author Contributions

AZ and SE carried out the electrophysiological and calorimetrical studies, and conducted single channel analysis. VY and IY designed, coordinated, and performed synthesis of the compounds. ZS took part in the discussion and experimental design. OO wrote the manuscript and contributed to the design and execution of the project. All authors contributed to the article and approved the submitted version.

## Conflict of Interest

The authors declare that the research was conducted in the absence of any commercial or financial relationships that could be construed as a potential conflict of interest.
