# Supplementary material for: 1,3-Thiazine, 1,2,3,4-Dithiadiazole, and Thiohydrazide Derivatives Affect Lipid Bilayer Properties and Ion-Permeable Pores Induced by Antifungals
Source: Front Cell Dev Biol. 2020 Jun 30;8:535. doi: 10.3389/fcell.2020.00535 (PMC7339130; doi:10.3389/fcell.2020.00535)
Supplement: Supplementary file 1 [file Data_Sheet_1.PDF]

Supplementary Material

1.1 Supplementary Figures

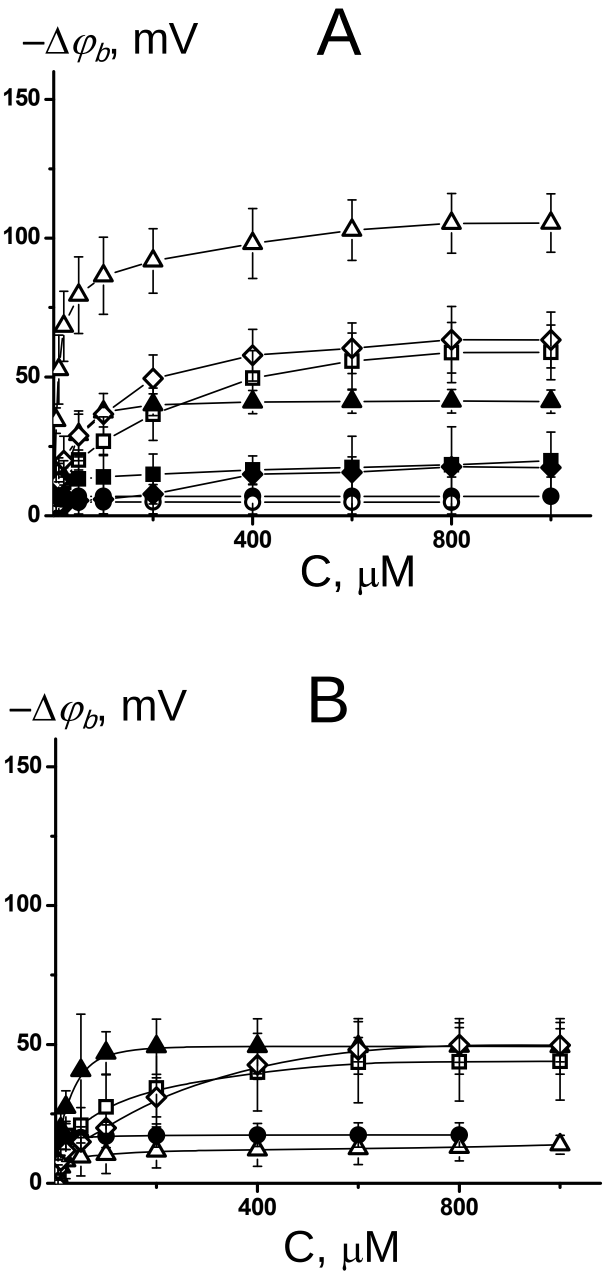

Supplementary Figure 1S. Dependence of the decrease in the boundary potential of the membrane ($-\Delta\varphi_b$) on the concentration of C1 (■), C2 (□), C3 (●), C4 (○), C5 (▲), C6 (Δ), C7 (♦), and C8 (◊). The membranes were composed of POPC (**A**) or POPC/POPG (50/50 mol%) (**B**) and bathed in 0.1 M KCl, pH 7.4. V = 50 mV.

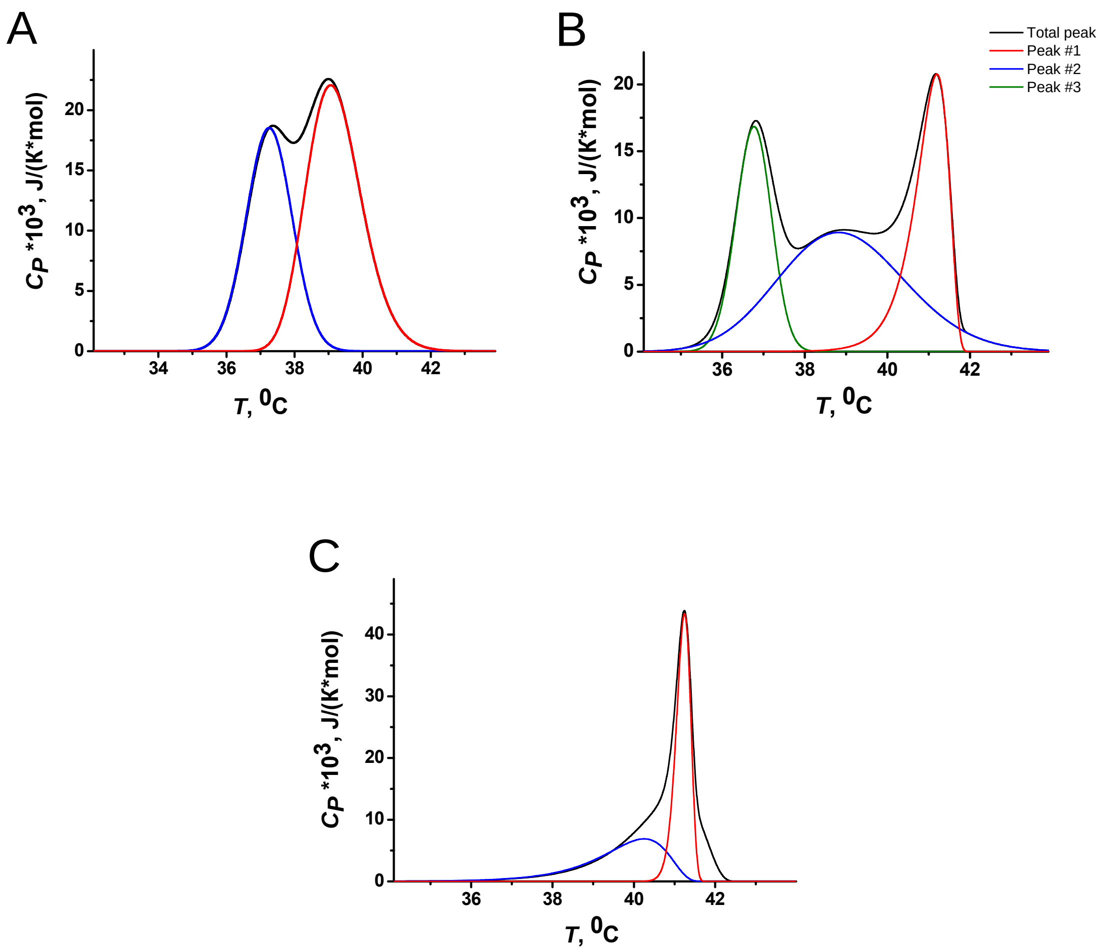

Supplementary Figure 2S. Deconvolution analysis of the main-transition peak of DPPC in the presence of C3 (**A**), C4 (**B**), and C6 (**C**) at the lipid:compound molar ratio of 10:1. The approximation parameters are summarized in TABLE 1S.

1.2 Supplementary Tables

Supplementary Table 1S. The physicochemical properties of the tested 1,3-thiazine, 1,2,3,4-dithiadiazole, and thiohydrazide derivatives: $LogD_{o/w}$ – the logarithm of octanol/water distribution coefficient at pH 7.4 predicted by Chemicalize (ChemAxon), μ – molecule dipole moment calculated by HyperChem 7.0 (Hypercube, Inc., Gainesville, FL, USA) using the semi-empirical MNDO.

compound	**$LogD_{o/w}$**	**μ, D**
C1	-1.02*	3.46
C2	$-0.36^{\$}$	6.07
C3	3.55	$1.03^{@}$
C4	3.98	$2.75^{\#}$
C5	$2.78^{\&}$	5.10
C6	4.10	5.72
C7	1.41	7.19
C8	3.37	6.81

*$LogD_{o/w}$ of acetyl-4-hydroxy-2H-1,3-thiazine
$^{\$}LogD_{o/w}$ of 5-[N-(2-aminophenyl)ethanimidoyl]-4-hydroxy-2H-1,3-thiazine
$^{\&}LogD_{o/w}$ of 2-(benzylsulfanyl)benzohydrazide
$^{@}$μ of 3-benzyl-5-phenyl-3H-1,2,3,4-dithiadiazole
$^{\#}$μ of 3-(2-methylphenyl)-5-phenyl-3H-1,2,3,4-dithiadiazole

Supplementary Table 2S. Deconvolution analysis of the main-transition peak of DPPC in the presence of C3, C4, and C6 at the lipid:compound molar ratio of 10:1.

compound	***№ of peak***	**T_m, °C**	**$T_{1/2}$, °C**
C3	***1***	39.0	2.0
	2	37.2	1.6
C4	***1***	41.1	1.0
	2	38.8	3.6

	3	36.8	1.0
C6	***1***	41.3	0.5
	2	40.2	1.9

Supplementary Table 3S. The parameters characterized the thermotropic behavior of DPPG in the presence of C1, C3, C6, and C8 at the lipid:compound molar ratio of 10:1: ΔT_p – the changes in the maximum temperature of the pretransition, ΔT_m – the changes in the maximum temperature of lipid melting; $\Delta T_{1/2}$ – the changes in the half-width of the main peak; $\Delta\Delta H$ – the enthalpy changes of the main phase transition.

compound	***DSC parameters***			
	**ΔT_p, °C**	**-ΔT_m, °C**	**$\Delta T_{1/2}$, °C**	**-$\Delta\Delta H$, kcal/mol**
C1	0	0	0	2 ± 2
C3	–*	0.6 ± 0.2	0.7 ± 0.1	8 ± 2
C6	–*	0.8 ± 0.2	0.5 ± 0.1	9 ± 3
C8	–*	0.4 ± 0.1	0.8 ± 0.1	7 ± 2

* – pretransition is suppressed. The temperatures of the pretransition and main transition of untreated DPPG are approximately 34.3 ºC and 41.0 ºC, respectively, with the half-width of the main peak of approximately 0.5 ºC. These data are in a good agreement with the results of Wang et al. (1994).

1.3 Supplementary References

Wang, P.Y., Lu, J.Z., Chen, J.W., Hwang, F. (1994). Interaction of the interdigitated DPPG or DPPG/DMPC bilayer with human erythrocyte band 3: differential scanning calorimetry and fluorescence studies. Chem. Phys. Lipids. 69(3), 241-249. doi:10.1016/0009-3084(94)90005-1.
